# Progress in the study of antibody-drug conjugates for the treatment of cervical cancer

**DOI:** 10.3389/fonc.2024.1395784

**Published:** 2024-06-06

**Authors:** Congcong Zhai, Yan Cui, Ling Guo, Cixiang Chen, Yanfang Song, Jinghua Zhong, Yili Wang

**Affiliations:** ^1^ Department of Oncology, Gannan Medical University, Ganzhou, China; ^2^ Department of Oncology, First Affiliated Hospital, Gannan Medical University, Ganzhou, China; ^3^ Department of Oncology, Bengbu Medical University, Lu’an, China; ^4^ Gannan Innovation and Translational Medicine Research Institute, Gannan Medical University, Ganzhou, China

**Keywords:** antibody-drug conjugates, cervical cancer, targeted therapy, clinical research, drug development

## Abstract

Cervical cancer is the second most prevalent malignancy affecting women’s health globally, and the number of morbidity and mortality from cervical cancer continues to rise worldwide. The 5-year survival rate of patients with recurrent or metastatic cervical cancer is significantly reduced, and existing treatment modalities have low efficacy and high adverse effects, so there is a strong need for new, effective, and well-tolerated therapies. Antibody-drug conjugates (ADCs) are a new targeted therapeutic modality that can efficiently kill tumor cells. This review aims to summarize the composition, research, and development history and mechanism of action of ADCs, to review the research progress of ADCs in the treatment of cervical cancer, and to summarize and prospect the application of ADCs.

## 1 Introduction

Cervical cancer is the second most common malignancy affecting women’s health globally (1, 2). There are more than 500,000 new cases of cervical cancer and about 250,000 deaths from cervical cancer each year, of which 80% occur in developing countries (3, 4). Patients with the International Federation of Obstetrics and Gynecology (IFOG) stage IB-IIA are usually treated with either surgery or radiotherapy and have a better prognosis, with a 5-year survival rate of 90%. For patients with IIB-IVA, simultaneous radiotherapy is given, and the 5-year survival rate is 57.1% (5). However, 11–64% of these patients will recur, and once recurrence or metastasis occurs, the 5-year survival rate decreases to 16.8% (6). Bevacizumab has been found to have a role in recurrent metastatic cervical cancer. In addition, tumor immunotherapy has led to an increase in the survival rate of patients with cervical cancer (7, 8). For patients with recurrent metastatic cervical cancer, pembrolizumab, in combination with chemotherapy, with or without bevacizumab, is the new first-line therapy for PD-L1-positive patients (9). With the emergence of drug resistance and adverse effects of immunosuppressive agents, there is an urgent need for an effective and well-tolerated new treatment to change the traditional treatment modality for cervical cancer, as well as to propose new first- and second-line treatments for patients with recurrent and metastatic cervical cancer. Antibody-drug conjugates (ADCs) consist of a monoclonal antibody targeting a tumor-specific or tumor-associated antigen coupled with a specific number of small-molecule payloads via a linker, which releases payload directly into the tumor cells and the tumor microenvironment, thereby efficiently killing and destroying the tumor cells (10). The treatment of ADCs in cervical cancer has become a hot issue in recent years. This review aims to summarize the composition, research, and development history, as well as the mechanism of action of ADCs, to review the research progress of ADCs in the treatment of cervical cancer, and to summarize and prospect the application of ADCs.

## 2 ADCs

ADCs utilize the cytotoxicity of the payload to kill tumor cells efficiently; the coupling effect of the linker effectively reduces the off-target toxicity of the payload to differentiate them from general monoclonal antibodies and chemotherapeutic drugs, thus achieving the purpose of targeted therapy, efficiency, and toxicity reduction (11).

### 2.1 Research process

**Figure 1:**
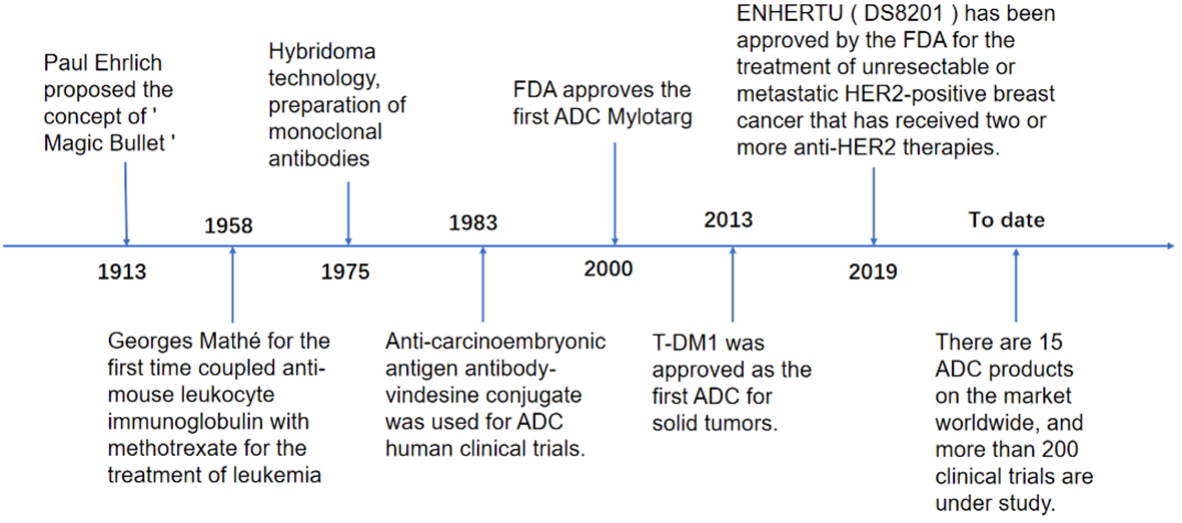
The research process of ADCs.

As early as 1913, Paul Ehrlich, a German scholar, proposed the concept of the “Magic Bullet,” i.e., selective delivery of toxic drugs to target cells. With the continuous development of science and technology, this concept has become a reality, and breakthroughs are constantly being made. In 1958, immunologist Georges Mathé first used anti-mouse leukocyte immunoglobulin and methotrexate to treat leukemia. In 1975, molecular biologists Georges J.F. Köhler and César Milstein created hybridoma technology based on natural hybridization technology and made the first hybridoma production based on natural hybridization. Technology to create hybridoma technology and prepare the first monoclonal antibody, 1983 First human clinical trial of an ADC using an anti-carcinoembryonic antigen antibody-vincristine coupling, 2000 FDA approval to market the first ADC, gitolizumab (Mylotarg), 2013 T-DM1 approved as the first ADC for solid tumors, 2019 ENHERTU (DS8201) approved by the FDA for the treatment of unresectable or metastatic human epithelial growth factor receptor-2 (HER-2) positive breast cancer that has received two or more anti-HER-2 therapies, 15 ADCs products on the market globally by 2023, and more than 200 clinical trials underway (12–14) (**Figure 1**).

### 2.2 Composition of ADCs

#### 2.2.1 Antibody

The successful development of ADCs depends on selecting appropriate target antigens, which are critical determinants of ADCs’ efficacy. ADCs take advantage of the differences in protein expression between cancer cells and normal cells to select appropriate target antigens (15). ADCs’ antibodies and their target antigens should satisfy the following conditions: a) The target antigens should be expressed homogeneously on the surface of the tumor cells, with little or no expression on normal tissues. b) The antibodies should bind to the target antigens with high affinity. Antigens with high-affinity binding can be endocytosed by cells after binding and further release the cytotoxin in tumor cells to play a role. c) The target antigen should have the characteristics of circulating on the surface of tumor cells, being relatively stable and non-secretory, which improves tumor targeting and reduces off-targeting possibilities to minimize the side effects. d) The antibody should have the characteristics of high specificity, low immunogenicity, long half-life, and good stability (16). The current interest of researchers in target antigens is not only focused on antigens expressed on tumor cells, but there is also a growing interest in antigens present in the tumor microenvironment, including antigens in the neovascular system, subendothelial extracellular matrix, and tumor stroma. Since all tumor cells’ survival depends on angiogenic and stromal factors, ADCs targeting these tissues may have broader efficacy (15). Among the known targets, HER-2 and Trophoblast Cell-Surface Antigen-2 (Trop-2) are two ideal targets for ADCs, which are specifically expressed in a variety of tumor cells (17).

Antibodies currently used in the development of ADCs are almost exclusively of the IgG class. There are four subclasses of IgG (IgG1, IgG2, IgG3, and IgG4) (**Table 1**). All subclasses have been used in clinical trials of ADCs except for IgG3, which has a low half-life in serum and is not used in ADCs (18, 19). Among them, IgG1 has high serum stability, a strong ability to immobilize complement, and a high binding affinity to target antigens, making it the most commonly used subclass in the design of ADCs. Compared with IgG1, IgG2 and IgG4 have comparable serum stability to IgG1, but both have low or no complement immobilization ability and far less binding affinity to target antigens than IgG1, so they are not used as optimal antibodies. However, IgG2 antibodies have been gradually used in the clinic because of their ability to form covalent dimers, and IgG4 antibodies have been used in the clinic because of their ability to form new heterodimeric bispecific monoclonal antibodies (14, 15, 20–22). First-generation ADCs used mouse monoclonal antibodies, which had the drawbacks of high immunogenicity, poor efficacy in humans, and short serum half-life. Therefore, second-generation ADCs use mouse/human chimeric monoclonal antibodies, which reduce immunogenicity and extend drug half-life. Most ADCs are currently approved for clinical use or are under development and use humanized or human monoclonal antibodies, which have strong antigenic affinity and specificity, long serum half-life, and minimal immunogenicity (14, 21). ADCs targeting cervical cancer are almost exclusively IgG1 antibodies; for example, Tisotumab Vedotin (TV) is an antibody that targets tissue factor (TF) as the target antigen, and its antibody is a fully humanized IgG1-kappa monoclonal antibody (23). ADCs targeting HER-2 also consist of human monoclonal IgG1 antibodies (24). ADCs against cervical cancer are almost exclusively IgG1 antibodies.

**Table 1:** Characterization of different IgG subtypes.

IgG subtype	t1/2(d)	ADCC	CDC	Fcγ affinity	stability
IgG1	21	+++	++	high	strong
IgG2	21	+/-	+	low	weak
IgG3	7	++	+++	high	weak
IgG4	21	+/-	–	medium	weak

+++, ++, +, – indicates intensity level of effect.

#### 2.2.2 Payload

The first generation of ADCs used conventional payloads such as doxorubicin, methotrexate, mitomycin, fluorouracil, and periwinkle alkaloids. At the same time, these chemotherapeutic agents had low potency, with half inhibitory concentration (IC50) values in the micromolar range, lack of selectivity, and poor accumulation in target cells, resulting in relatively low efficacy of early ADCs. Subsequently, more potent payloads were used with IC50 values in the subnanomolar range, which had desirable properties such as high plasma stability, low immunogenicity, small molecular weights, and long half-lives (25). Commonly used cytotoxins can be divided into three classes: microtubule protein inhibitors such as orlistat in and adenosine; DNA synthesis inhibitors such as kanamycin, domicicicastin, and pyrrolobenzodiazepines; topoisomerase inhibitors and RNA polymerase II inhibitors such as α-oligo muscarinic acid and comedones derivatives (26). Currently, potent microtubule protein inhibitors such as orlistatins and highly active cytotoxic drug-carrying DNA topoisomerase inhibitors are commonly used as payloads in treating cervical cancer ADCs.

#### 2.2.3 Linker

The ADCs’ linkers connect the antibody to the payload. The ideal linker should ensure that it is stable enough in the bloodstream not to divide prematurely, leading to off-target toxicity. It can also divide rapidly to release the payload once it enters the tumor cell for internalization. It is the critical component that determines the toxicity and efficacy of ADCs (27).

**Figure 2:**
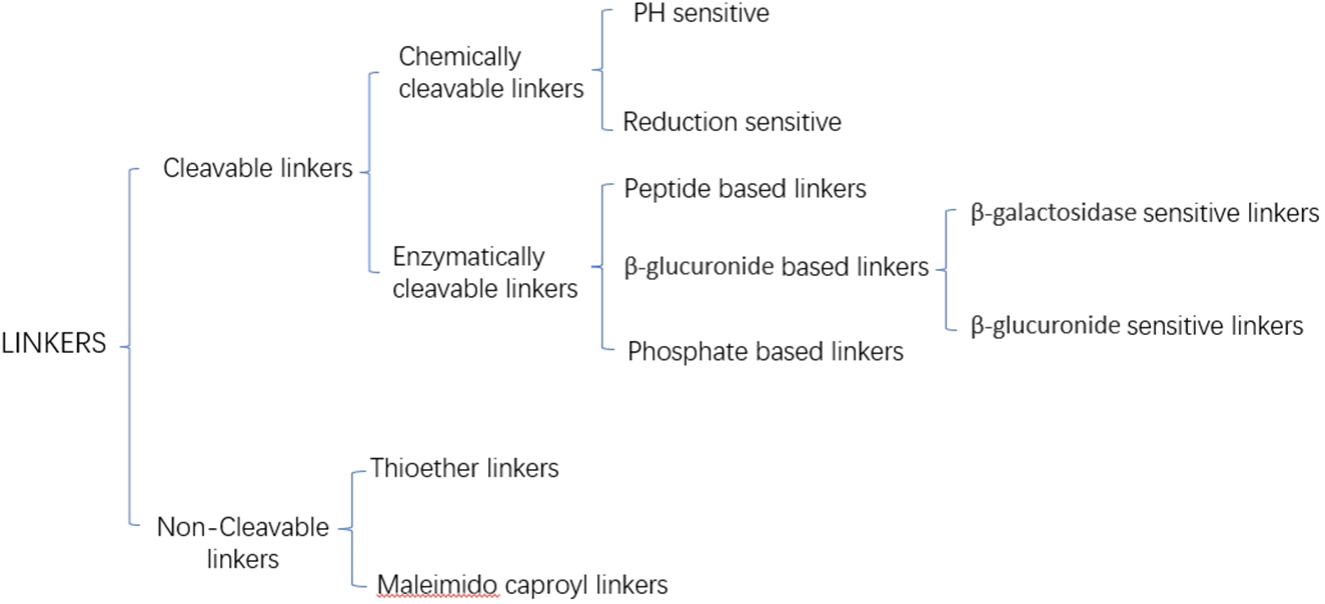
Subcategory of linkers.

Current linkers are categorized into cleavable and non-cleavable linkers based on their release mechanism (**Figure 2**) (28). Non-cleavable linkers form a fundamental bond between a cytotoxic drug and an amino acid residue of an antibody, e.g., Thioether linkers, and Maleimido caproyl linkers. In contrast, cleavable linkers can be categorized into chemically cleavable and enzymatically cleavable linkers based on the cleavage mode. Chemically cleaved linkers are cleaved based on the different environments of blood and cytoplasm, e.g., stilbene linkers remain stable in a neutral blood environment. Still, they will be cleaved to release cytotoxins in an acidic cytoplasm. Disulfide linkers depend on the difference in glutathione concentration inside and outside the cell and release cytotoxins through redox reactions. The most widely used of the enzyme cleavage linkers is the peptide linker, which utilizes lysosomal proteases highly expressed in tumor cells to enzymatically cleave the amide bonds of the linker to release cytotoxins (29).

The mode of antibody-payload coupling significantly impacts the biological activity, tolerance, and drug stability of ADCs. There are two types of coupling methods: targeted and non-targeted coupling. Early ADCs mainly by the amino group (- NH2) of lysine and the sulfhydryl group (- SH) of cysteine. The non-specific coupling relies on lysines, and typically, there are more than 80 lysines on the antibody, but only 20 or so sites suitable for coupling, resulting in a randomized reaction and, therefore, a lack of homogeneity in the product. In addition, lysine coupling may alter the amount of charge on the antibody itself, thus affecting the stability of the antibody, so there should not be too much payload in this coupling method (30). Targeted coupling relies on cysteines, which are relatively undistributed in the antibody, and the monoclonal antibody contains 16 pairs of disulfide bonds, of which 12 pairs of intrachain disulfide bonds and four pairs of interchain disulfide bonds. A reducing agent acts upon the interchain disulfide bonds to create eight cysteine binding sites. By this coupling method, drug-to-antibody ratio (DAR), i.e., the number of drug molecules attached to a monoclonal antibody, is obtained for ADCs with a molecular number of 0, 2, 4, 6, or 8 (31). Using fixed-point coupling in the new generation of ADCs improves their stability (29, 32). If too little payload is coupled, it reduces its biological activity, and too much increases the clearance of ADCs from the bloodstream while affecting the effective binding of antigens and antibodies, in general, the ideal DAR is 2–4 (33).

### 2.3 The anti-tumor mechanism of ADCs

**Figure 3:**
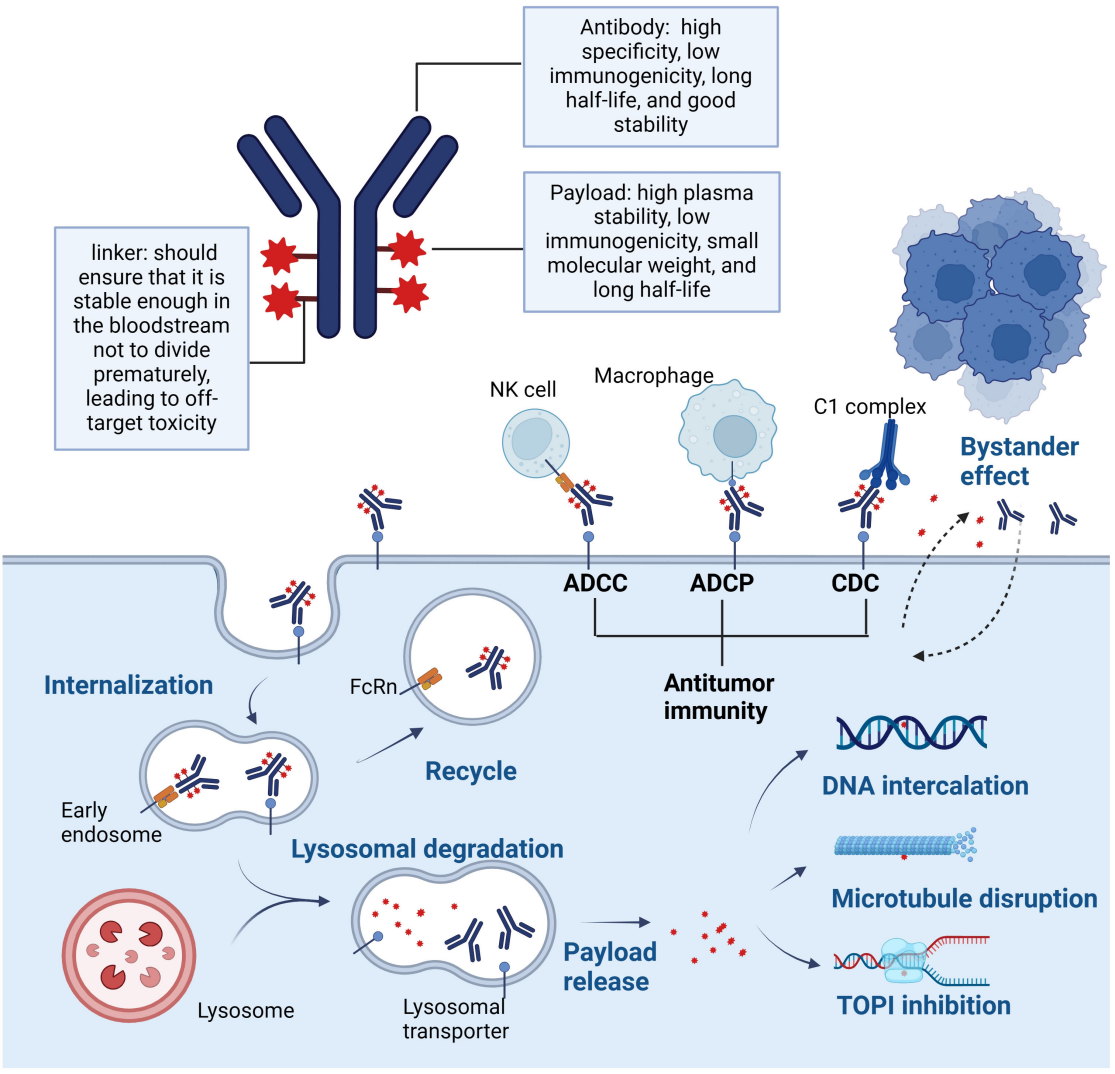
Anti-tumor mechanism of ADCs.

To improve bioavailability, ADCs are mainly administered intravenously. After ADCs enter the blood circulation, they specifically bind to the antigen on the surface of tumor cells to form ADC-antigen complexes, forming early endosomes containing ADC-antigen complexes, which enter tumor cells through endocytosis (34). The decomposition of ADCs with cleavable linkers is mainly accomplished in the nuclear endosomes. ADCs with non-cleavable linkers must be hydrolyzed in lysosomes to release cytotoxins, which cause apoptosis by inducing DNA damage or inhibiting microtubule polymerization (21). Some hydrophobic cytotoxic drugs called the bystander effect, can also diffuse through the cell and exert killing activity on neighboring tumor cells and surrounding stromal tissues. In addition, the anti-tumor mechanism of ADCs has been associated with complement-dependent cytotoxicity (CDC), antibody-dependent cell-mediated cytotoxicity (ADCC), antibody-dependent cellular phagocytosis (ADCP), and other mechanisms (35) (**Figure 3**).

### 2.4 Mechanisms of ADCs resistance

The resistance mechanisms to ADCs are complex and are influenced by various factors, which may arise either from each component of the ADCs or from one of the pathways in the mechanism of action of the ADCs (36). Down-regulation and mutation of antigens are among the main reasons for triggering resistance to ADCs. Ideally, the cell surface expression of the target antigens should be free from down-regulation by the effects of repetitive stimuli during the treatment. Therefore, the selection of appropriate target antigens is critical for ADCs (37). There are many other potential mechanisms of ADCs resistance. Examples include disruption of lysosomal function, blocked payload transport, payload-related resistance, etc. The impact of ADCs resistance on therapies acting on the same target or ADCs treatment through the action of the same cytotoxic drug is still unknown. With rapid advances in protein engineering, innovative immunostimulatory ADCs using interferon gene agonists, toll-like receptor agonists, and antibody chemokine couplers are being developed to give ADCs the cytotoxicity of the coupler while enhancing the immune response for dual antitumor effects (38).

## 3 Application of ADCs in the treatment of cervical cancer

An increasing number of ADCs are targeting highly expressed targets in cervical malignancies, and a large number of clinical trial studies are currently underway **Tables 2**, **3**.

**Table 2:** FDA-approved marketed and -in clinical studies for cervical cancer ADCs.

Target	Drug name	Payload	Linker	Clinical state	Main study	Research stage	Indications
TF	TV	MMAE	MC-VC-PABC	2021.09 Approved	InnovaTV 201	I/II	Recurrent metastatic cervical cancer
					InnovaTV 204	II	
					InnovaTV 206	I/II	
					InnovaTV 205	I/II	
					InnovaTV 301	III	
HER-2	T-DXd	Dxd	MC-GGFG	in research	DESTINY-PanTumor02	II	HER-2-positive advanced cervical cancer
	RC48	MMAE	MC-VC-PABC	in research	C018	II	
Trop-2	SG	SN-38	CL2A	in research	EVER-132-003	II	Recurrent metastatic cervical cancer
Nectin-4	9MW-2821	MMAE	IDconnect	in research	CTR20220106	I/II	
Claudin18.2	/						Cervical adenocarcinoma

**Table 3:** Summary of experimental data of ADCs related research.

Main study	Number of participants	ORR	mDOR(m)	mPFS(m)	mOS(m)
InnovaTV 201	27	22%	6.0	/	/
InnovaTV 204	101	24%	8.3	4.2	12.1
InnovaTV 206	17	29.40%	7.1	3.1	11.4
InnovaTV 205(1L TV+Pembro)	32	40.60%	NR	5.5	NR
InnovaTV 205(2L/3L TV+Pembro)	34	38.20%	14	5.6	15.3
InnovaTV 205(1L TV+Carbo)	33	54.50%	8.6	6.9	NR
InnovaTV 301	253	17.80%	5.3	4.2	11.5
DESTINY-PanTumor02	40	50%	9.8	7	13.4(All people)
C018	25	36.40%	5.52	4.37	NR
EVER-132-003	18	50%	9.2	8.1	/
CTR20220106	40	40.54%	NR		

NR, not reached.

/, not available.

### 3.1 Targeting TF

On September 20, 2021, TV, an ADC co-developed by Seagen and Genmab, was approved for marketing by the FDA (39). TV is a fully humanized IgG1-kappa monoclonal antibody coupled to 4 molecules of monomethyl auristatin E (MMAE), a potent microtubule protein inhibitor, via a protease cleavable linker with TF as the target antigen (40). TF is a transmembrane glycoprotein mainly expressed in vascular endothelial subdural cells. Under normal physiological conditions, the central role of TF is to act as a physiological initiator of the exogenous coagulation pathway, and activation of the coagulation cascade reaction occurs after disruption of the vessel wall due to injury or after up-regulation of tissue factor by monocytes under inflammatory conditions. Binding to coagulation factor VII (FVIIa) induces phosphorylation of protein kinases regulated by extracellular signals, followed by activation of protease-activated receptor 2, leading to an intracellular signaling cascade that results in the production of pro-angiogenic factors, cytokines, and adhesion molecules that promote tumor growth, angiogenesis, and tumor metastasis (33). TF is lowly expressed in normal tissues but is overexpressed in several solid tumors, including cervical cancer, and is closely related to the poor prognosis of the disease; therefore, TV has become an ideal target for the development of ADCs (41, 42). TV can exert its antitumor effects through multiple mechanisms. TV binds to TF and then translocates to the lysosome, where the linker is cleaved enzymatically, releasing MMAE, which binds to microtubule proteins and kills the target cells; MMAE also diffuses into the tumor microenvironment and exerts a bystander effect; TV can activate macrophages, natural killer cells, dendritic cells, and other immune cells to exert antitumor effects; in addition, TV can inhibit the activation of TF-related signaling pathway caused by FVIIa, which further enhances the antitumor effects (43, 44).

InnovaTV 201 is the first phase I/II, open-label, dose-escalation, and dose-expansion clinical trial of TV (NCT02001623) (45) designed to evaluate the safety, tolerability, pharmacokinetic profile, and antitumor activity of TV in a variety of recurrent, progressive, and metastatic solid tumors. Inclusion criteria: age ≥18 years; having recurrent, advanced, or metastatic tumors, which include ovarian, cervical, endometrial, bladder, prostate, esophageal, head and neck squamous cell, or non-small cell lung cancers; an Eastern Cooperative Oncology Group (ECOG) score of 0 to 1; and inability to receive standard therapy or patients who relapsed after receiving standard treatment; and unlimited tissue factor expression. In the dose-escalation phase, 27 patients received 0.3 to 2.2 mg·kg-1 (once every three weeks, iv) TV monotherapy in a conventional 3 + 3 design. It was found that patients receiving 2.2 mg·kg-1 TV experienced dose-limiting toxicities, including grade 3 type 2 diabetes, mucositis, and neutropenic fever. In contrast, a predictable pharmacokinetic profile and manageable toxicity were noted at the 2.0 mg·kg-1 dose, and therefore, 2.0 mg·kg-1 was determined to be the recommended dose for phase II. Patients were treated at the recommended Phase II dose during the dose extension phase. The primary endpoint was the occurrence of adverse events. The results showed that 55 patients with recurrent or metastatic cervical cancer treated with TV had an Objective Response Rate (ORR) of 22% and a Median During Of Response (mDOR) of 6.0 months. This study suggests that TV demonstrates preliminary antitumor activity in previously treated pan-tumor patients, especially those with recurrent or metastatic cervical cancer.

**Figure 4:**
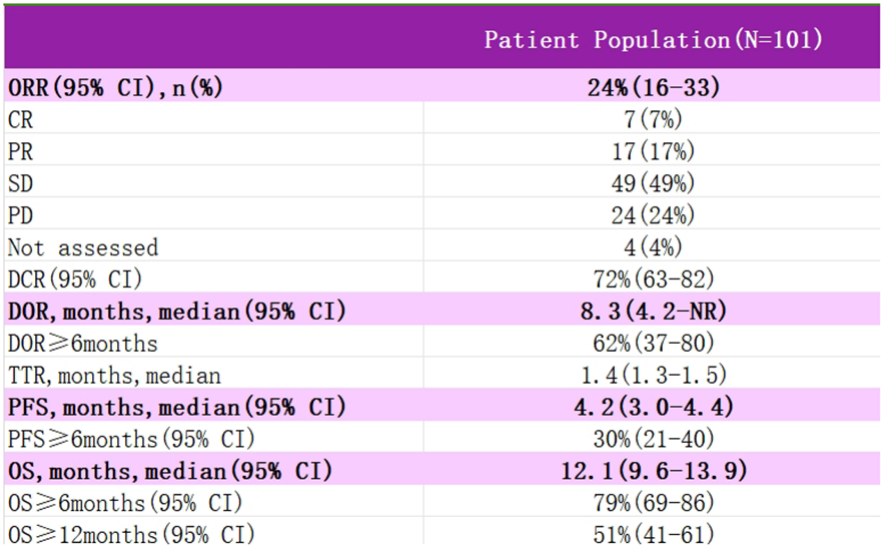
Test data for InnovaTV 204.

InnovaTV 204 was a multicenter, open-label, single-arm, phase II clinical study (NCT03438396) (46) designed to evaluate TV’s efficacy and safety study for recurrent or metastatic cervical cancer (**Figure 4**). The study included 102 patients with recurrent or metastatic cervical cancer (histologic type 68% squamous cell carcinoma, 27% adenocarcinoma, and 5% adenosquamous carcinoma), ECOG score 0–1, and age ≥18 years. Patients had received no more than two prior systemic regimens (including at least one platinum-containing chemotherapy), of which 54% had received prior cisplatin plus radiotherapy, 70% had received one last systemic treatment for recurrent or metastatic disease, 63% had received prior bevacizumab plus two-agent chemotherapy as first-line treatment, and 56% had not responded to the last systemic treatment. In this study, patients received TV (2 mg·kg-1 every three weeks, iv) monotherapy until disease progression or serious adverse events occurred. The median follow-up was 10.0 months. The results showed that patients treated with TV had an ORR of 24%, of which 7% achieved Complete Response (CR) and 17% Partial Response (PR), the Disease Control Rate (DCR) was 72%, 79% of patients had a lesion reduction from baseline, and the mTOR was 8.0%. Disease control rate (DCR) was 72%, 79% of patients had a reduction in lesions from baseline, mDOR was 8.3 months (higher than the current 2–6 months for single-agent chemotherapy), and median Overall Survival (mOS) was 12.1 months. Everyday treatment-related adverse events were alopecia, rhinorrhea, nausea, conjunctivitis, fatigue, and dry eye. Grade 3 or more treatment-related severe adverse events, including neutropenia, fatigue, ulcerative keratitis, and peripheral neuropathy, were reported in 28% of patients, and 13% experienced a severe adverse reaction, most commonly peripheral sensorimotor neuropathy and fever. In this study, 80 of the study subjects were assayed for tumor cell membrane TF expression, and the positivity rate was up to 96% (77/80), suggesting that TV can be widely used in patients with recurrent or metastatic cervical cancer; a total of 32 of the non-squamous cancer (adenocarcinoma, adenosquamous carcinoma) subjects in this study had an ORR of 24%, suggesting that TV is effective for the treatment of adenocarcinomas; the median time to response was 1.4 months, suggesting potential antitumor activity in the first two treatment The median response time was 1.4 months, indicating potential antitumor activity within the first two treatment cycles, and TV is a fast-responding drug; after previous use of bevacizumab or chemotherapy resistance, TV can still show antitumor activity when used again. In September 2021, the FDA granted accelerated approval under the InnovaTV 204 trial accelerated approval of TV for patients with recurrent or metastatic cervical cancer whose disease progressed during or after chemotherapy.

InnovaTV 206 was a Phase I/II clinical trial (NCT03913741) to evaluate TV’s safety, efficacy, and pharmacokinetics in Japanese patients with solid tumors, including cervical cancer. The enrollment criteria were essentially the same as those of the InnovaTV 204 study, and the trial was divided into two parts; the first part was a drug dosage safety exploration, which determined that the standard dose of TV in the phase II study was two mg·kg-1 once every three weeks; a total of 17 subjects were enrolled in the phase II study, with nine squamous cancers, eight adenocarcinomas, and 8 (47.1%) who had recurrence of the prior treatment in combination with bevacizumab. The 17 subjects were independently reviewed for CR, PR, Stable Disease (SD), Progressive Disease (PD), and non-evaluable in 0, 5 (29.4%), 7 (41.2%), 3 (17.6%), and 2 (11.8%), respectively, and the final ORR (CR + PR) was determined to be 29.4% (5/17), and DCR (CR + PR + SD) was 70.6% (12/17); mean time to onset of efficacy was 1.2 months for TTR and 7.1 months for DOR; PFS was 3.1 months and PFS ≥ 6 months in 26.1% of subjects; and OS was 11.4 months, ≥ six months and ≥ 12 months in 81.6% and 25.7% of subjects. The treatment-related adverse reactions reported in the InnovaTV 206 study were generally consistent with InnovaTV 204. However, due to the small sample size of this study, the association between TF expression and TV efficacy is inconclusive.

InnovaTV 205 is a global, multicenter, open phase I/II clinical study (NCT03786081) (47) designed to evaluate the safety and antitumor activity of TV alone or in combination with bevacizumab, pembrolizumab, or carboplatin in patients with recurrent or metastatic cervical cancer who are untreated and previously treated. The dose-escalation phase consisted of three cohorts (A, B, and C) to determine the maximum tolerated dose of TV in combination therapy and the recommended dose for the next phase, and the dose-expansion phase consisted of five expansion cohorts (D, E, F, G, and H).2021 At the European Society of Medical Oncology meeting, the investigators presented interim data from two of these cohorts (1): In Cohort D (TV+carboplatin), 33 patients with recurrent or metastatic cervical cancer who had not undergone systemic therapy were treated with a combination of TV (2 mg-kg-1 every three weeks, iv) and carboplatin (AUC=5, every three weeks, iv), with an ORR of 55% at a median follow-up of 4.8 months (n=18), including 2 CRs and 16 PRs, with an mDOR of 5.6 months, and a median time to remission (mTTR) of 5.6 months. The mTOR was 5.6 months, the median time to remission (mTTR) was 1.4 months, and the median progression-free survival (PFS) was 6.9 months (2). In Cohort F (TV+papolizumab), 35 patients with recurrent or metastatic cervical cancer who experienced disease progression during or after 1 or 2 prior systemic therapies were treated with a combination of TV (2 mg·kg-1 every three weeks, iv) and papolizumab (200 mg every three weeks, iv), of whom 81.5% were PD-L1-positive, and 74.3% had received prior first-line therapy and 51.4% had received bevacizumab. At a median follow-up of 10.2 months, the ORR was 35%, including 2 CRs and 10 PRs, with an mTOR of 1.4 months and mPFS of 5.6 months. In conclusion, TV combined with carboplatin as a first-line treatment option for patients with recurrent or metastatic cervical cancer who have not received prior systemic therapy, and TV combined with pembrolizumab as a second- or third-line therapy for patients with recurrent or metastatic cervical cancer, both have encouraging and durable antitumor activity. The InnovaTV 205 study design covers first- and backline therapy for the systemic treatment of recurrent metastatic cervical cancer, including monotherapy and combinations, with a comprehensive layout that may change the treatment landscape for recurrent metastatic cervical cancer if the results are positive.

**Figure 5:**
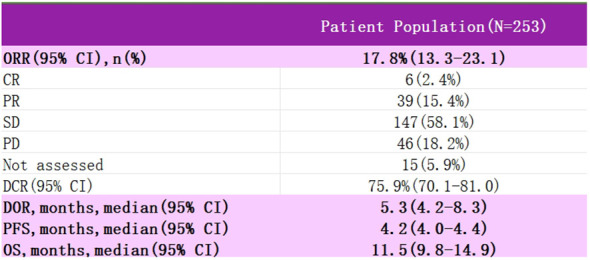
Test data for InnovaTV 301

InnovaTV 301 was a phase III clinical trial (NCT04697628) (48) that enrolled patients with recurrent metastatic cervical cancer who had received prior systemic therapy, including conventional two-agent chemotherapy with paclitaxel and platinum, with the majority of the patients also having received bevacizumab, and even a subset of the patients having received immunotherapy (**Figure 5**). The study then randomized patients into two groups, one receiving TV and the other standard chemotherapy (e.g., topotecan, vincristine, gemcitabine, irinotecan, pemetrexed). At the 2024 SGO meeting, the most recent data from the study were presented: among patients with recurrent metastatic cervical cancer who had received prior therapy, TV showed statistically significant and clinically meaningful improvement in efficacy; the risk ratio for OS was 0.70, and the risk of death was reduced by 30%. The ORR advantage of the TV group was consistent regardless of TF expression. The safety of the TV was controllable and manageable. Based on these data, TV is a potential new standard of care for patients who have progressed after systemic therapy for 1L.

TV delivers the microtubule protein inhibitor MMAE directly to tumor cells for release by targeting tissue factors.TV has demonstrated promising and durable antitumor activity in ≥2 lines of treatment for recurrent/metastatic cervical cancer, with a tolerable safety profile and a survival benefit over chemotherapy.TV in combination with pembrolizumab or carboplatin ± bevacizumab has demonstrated promising efficacy. The use of TV for first-line treatment of cervical cancer is under investigation. Tissue factor expression is not limited to cervical cancer, and expansion of TV pan-tumor studies is expected.

### 3.2 Targeting HER-2

#### 3.2.1 T-DXd

T-DXd is a novel antibody-coupled drug that the FDA granted expedited drug review status in December 2016; the FDA granted accelerated approval for T-DXd as a second-line treatment option for unresectable or metastatic HER-2 positive breast cancer on December 20, 2019. T-DXd has been used in non-small cell lung cancer, gastric cancer, and cholangiocarcinoma with remarkable efficacy and safety (49). T-DXd is a HER-2 targeting drug consisting of a human monoclonal IgG1 produced with the same amino acid sequence as trastuzumab, an enzymatic peptide linker, and a highly active cytotoxic drug-carrying DNA topoisomerase I inhibitor called deruxtecan (DXd).T-DXd has a high level of DAR, which can be close to 8, which is 2 to 4 times higher than the already approved ADCs, helping to deliver more drugs to target cells. Higher loaded drug activity, 1000 times more potent than commonly used chemotherapeutic agents, and ten times more powerful than the active metabolite of irinotecan. The shorter drug half-life and high stability of the linker and the payload allow for faster excretion into the body and rapid reduction of DXd blood levels, helping to minimize adverse reactions. The high permeability of the payload enables it to exert a bystander-killing effect (50–52). HER-2 abnormalities are widespread in solid tumors, with an immunohistochemical (IHC) positivity rate of about 20% in gynecologic tumors, including an unexpectedly high HER-2 positivity rate in uterine carcinosarcoma (UCS) and a high HER-2 IHC scores of 3+, 2+ and 1+ were found in 25%, 35% and 40% of UCS cases, respectively. A Phase II, multicenter, single-arm, investigator-initiated trial evaluating the efficacy and safety of T-DXd in patients with advanced or recurrent HER2-overexpressing UCS who had received prior standard chemotherapy, demonstrated that T-DXd was effective in patients with UCS, regardless of HER-2 status. Safety toxicity is manageable with appropriate monitoring and treatment (53).

**Figure 6:**
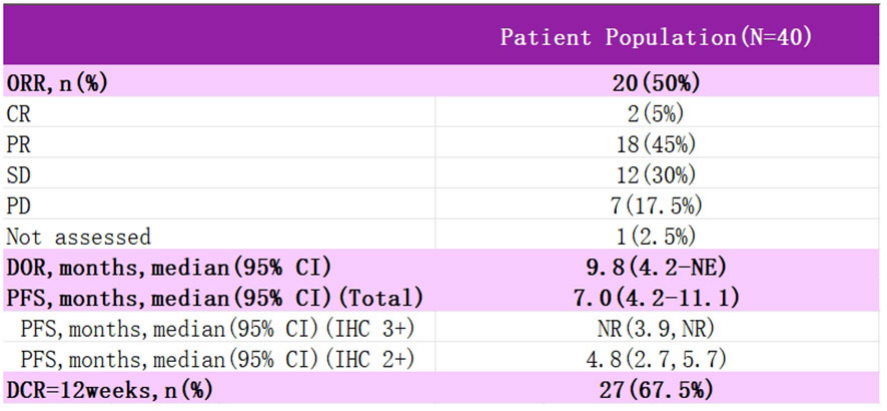
Test data for DESTINY-PanTumor02.

DESTINY-PanTumor02 is an open-label, multicenter, phase II trial (NCT04482309) (**Figure 6**) (54). It was designed to evaluate the efficacy and safety of T-DXd (5.4 mg·kg-1 every three weeks) after ≥ one systemic therapy or no alternative therapy in patients expressing HER2 (immunohistochemistry IHC 3+/2+ by local or central assay), locally advanced or metastatic disease patients with efficacy and safety. The primary endpoint was ORR, as assessed by the investigators. Secondary endpoints included DOR, DCR, PFS, OS, and safety. RESULTS: In the initial analysis, 267 patients were treated in seven tumor cohorts: endometrial, cervical, ovarian, bladder, biliary, pancreatic, and other. The median follow-up was 12.75 months. Among all patients, ORR was 37.1%, all cohorts responded, median DOR was 11.3 months, median PFS was 6.9 months, and median OS was 13.4 months. In patients with HER2 IHC 3+ expression (n=75), ORR was 61.3%, median DOR was 22.1 months, median PFS was 11.9 months, and median OS was 21.1 months. Grade≥3 drug-related adverse events were observed in 40.8% of the patients, and drug-related interstitial lung disease (ILD) was adjudicated in 10.5% of the patients, with three deaths. In all cervical cancer patients, ORR was 50%, mDOR 9.8 months, and PFS 7.0 months, with 75% ORR and PFS NR in IHC 3+ patients. The study suggests that T-DXd could be a potential treatment option for patients with HER2-expressing cervical cancer. Additional data from 2024 T-DXd demonstrated a clinically meaningful ORR in patients with HER-2-expressing gynecologic neoplasms. Regardless of prior, the safety profile of T-DXd was consistent with prior. These data support that T-DXd could be a potential treatment for patients with HER-2-expressing gynecologic tumors after progression on prior therapy.2024 The new NCCN Cervical Cancer Guidelines have added a new recommendation for T-DXd to be included in the second-line treatment of HER-2-positive patients (targeting HER-2 IHC 2+ or 3+). T-DXd is available domestically and is supra-indicated for use in gynecologic oncology patients.

#### 3.2.2 RC48

RC48 contains the novel humanized anti-HER2 antibody Hertuzumab, linked to monomethylated fundic sparrow isoflavin E (MMAE) via a cleavable linker. Hertuzumab has a higher affinity for HER2 and more robust antibody-dependent cell-mediated cytotoxicity (ADCC) activity *in vitro* than Trastuzumab (55). A phase II clinical study of RC48-ADC in combination with radiotherapy, PD-1/L1 inhibitors, GM-CSF, and IL-2 for the treatment of patients with HER2-overexpressing advanced solid tumors was performed. ENTRY CRITERIA: Patients with advanced solid tumors; confirmed HER2 expression (IHC 3+, 2+, or 1+); patients who have progressed on, or are intolerant of, standard therapy. Receive RC48-ADC (2.0 mg/kg d1 every three weeks) followed by 2–3 doses of HFRT (5–8 Gy) to one metastasis every other day during the treatment cycle, subcutaneous GM-CSF (200 mg d3–7) initiated at the end of radiotherapy, sequential IL-2 (2 million IU d8–12), and PD-1/L1 within one week after completion of radiotherapy inhibitor, RC48-ADC combined with PD-1/L1 inhibitor sequential cytokines for at least six cycles followed by continued maintenance therapy with PD-1/L1 inhibitors until disease progression or unacceptable toxicity. As of January 2023, 32 patients (6 with gynecologic tumors) were enrolled, with the primary endpoint being ORR. The study results showed an ORR of 66.7% for gynecologic tumors, and the remission rate for HER2 IHC1+ patients was similar to that of IHC2+-3+ patients, with ORRs of 43.8% and 30%, respectively.

### 3.3 Targeting Trop-2

Trop-2 was first found in human placental trophoblasts and is expressed in various normal tissues. Subsequently, various tumor cells were found to have high expression of Trop-2, thus attracting attention in tumor therapy (56). Sacituzumab govitecan (SG) is an ADCs consisting of a humanized anti-Trop-2 antibody, which is coupled to the active metabolite of irinotecan (SN-38) via a pH-sensitive hydrolyzable linker that supports bystander effects in the tumor environment (57). On February 3, 2023, the U.S. FDA approved SG for use in patients with unresectable, locally advanced or metastatic hormone-receptor (HR)-positive, HER-2-negative breast cancer with ≥2 lines of systemic therapy who have received prior endocrine therapy, making it the world’s first and only ADCs-class drug approved to target Trop-2. Previous studies have shown that Trop-2 targeted by SG is highly expressed in nearly 90% of cervical cancers and is highly expressed in different histological types of tumors, so the clinical study of SG for recurrent or metastatic cervical cancer has also been highly concerned (58).

**Figure 7:**
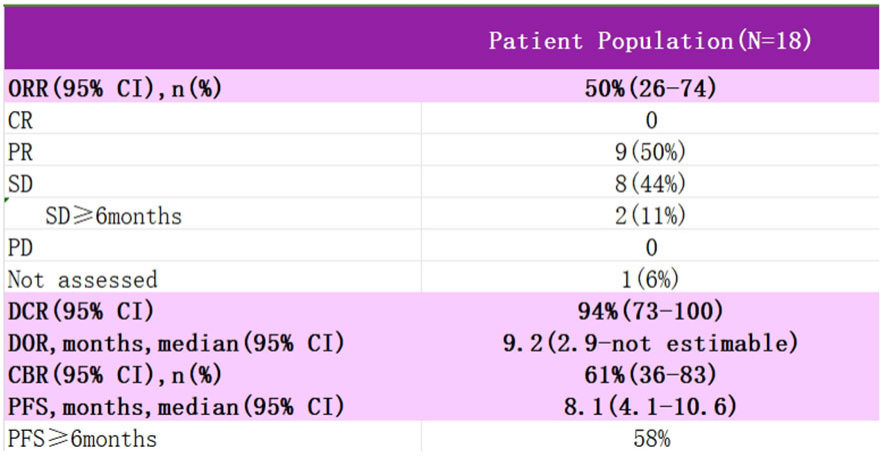
Test data for EVER-132-003.

Study EVER-132–003 is a multicenter, single-arm, multi-cohort clinical Phase II study using SG in the treatment of patients with a variety of solid tumors, enrolling patients with advanced cervical cancer who are resistant or intolerant to platinum and paclitaxel-based chemotherapeutic agents, who were treated with an intravenous infusion of SG 10 mg·kg-1 on days 1 and 8 of every 21 days, with the primary endpoint of the study being the investigator-assessed ORR (**Figure 7**). Data from an interim analysis of the study were reported at SGO 2024, with SG monotherapy in patients with recurrent metastatic cervical cancer showing outstanding anti-tumor activity with a 50% ORR and sustainable treatment benefit, as well as significant benefit in patients with prior immunotherapy, and no new safety signals were identified. For patients with recurrent metastatic cervical cancer, for whom the current treatment options are still minimal, SG is promising to be an ideal choice for future backline treatment, and we look forward to subsequent clinical studies to further evaluate the therapeutic value and long-term benefits of SG in patients with recurrent metastatic cervical cancer.

### 3.4 Targeting The Nectin cell adhesion protein 4

Nectin-4 is a member of the Nectin family, and unlike Nectin-1, 2, and 3, which are widely expressed in adult tissues, Nectin-4 is only expressed in the embryo and placenta, and its overexpression is associated with growth and invasiveness as well as a poor prognosis in a variety of tumors (59). Currently, there is only one ADC, Senfortumab vedotin (EV), approved for marketing for the second-line treatment of patients with uroepithelial carcinoma (60). In addition to EV, there are several clinically investigational Nectin-4 ADCs, including Mavis Biologics’ 9MW-2821, which consists of an anti-Nectin-4 antibody (MW282 mAb), the novel linker IDconnect, and the known MMAE. IDconnect is intended to be used in the treatment of uroepithelial carcinoma by cross-linking the antibody Fab and reduced cysteines in the hinge region to form site-specific disulfide bonds, thereby enabling the DAR to achieve a high degree of homogeneity of 4 (61). 9MW2821 was included in a cervical cancer cohort for the first time, which is the first exploration of an ADCs targeting Nectin-4 in the treatment of cervical cancer.

A clinical phase I/II trial of 9MW2821 in adult patients with recurrent or metastatic cervical cancer treated with 1 or 2 lines of therapy with or without bevacizumab showed a detection rate of 89.67% for Nectin-4 expression and 67.82% for Nectin-4 tumor cell staining intensity 3+ in the study’s cervical cancer expansion cohort as of September 25, 2023 In the expansion cohort, the detection rate of Nectin-4 expression was 89.67%, and the detection rate of Nectin-4 tumor cell staining intensity 3+ was 67.82%. Due to the high expression rate of Nectin-4 in the cervical cancer population, patients will not have to undergo too strict screening before treatment and thus have higher accessibility after entering the clinical application in the future.

### 3.5 Targeting Claudin18.2

Claudin proteins are critical structural proteins for intercellular tight junctions, and their isoform Claudin18.2 is expressed explicitly in differentiated gastric epithelial cells. It is hyperactivated during cellular malignancy and in pancreatic, esophageal, and colorectal cancers. Although the IHC interpretation criteria for Claudin18.2 need further standardization, preliminary clinical trials of drugs targeting Claudin18.2 have shown promising antitumor efficacy (62). Cervical adenocarcinoma is characterized by gastric pyloric epithelial differentiation, and almost 100% of patients with Cervical adenocarcinoma have positive Claudin18.2 expression. Therefore, although there are no published clinical data on drugs targeting Claudin18.2 in cervical cancer, this target deserves the attention of patients with Cervical adenocarcinoma (63).

## 4 Summarized and prospected

ADCs open a new era of “pan-tumor molecularly guided targeted therapy.” TV for cervical cancer has been approved by the FDA and recommended by NCCN guidelines and is expected to be launched in China as soon as possible. T-DXd has shown significant efficacy in HER-2 high expression cervical cancer, with a trend of benefit in ORR and PFS, and will be available in China in February 2023 for breast cancer indication. Breast cancer indication has been approved for marketing; HER-2-positive cervical cancer patients can be individualized for super-adaptation application. Vedicilimumab has promising efficacy in Her2-positive gynecological tumors, has been approved for marketing in gastric cancer and uroepithelial cancer indications in China, has a price advantage, and can be applied individually with super-indications in HER-2-positive cervical cancer patients. SG targeting Trop-2 has shown outstanding anti-tumor activity in the treatment of recurrent metastatic cervical cancer patients. Patients who have previously received immunotherapy can also benefit significantly, and no new safety signals have been found. For patients with recurrent metastatic cervical cancer who are still very limited in the current treatment choices, SG is very hopeful of becoming an ideal choice of backline treatment in the future. 9MW2821, which targets Nectin-4, has also shown surprising results in the treatment of recurrent metastatic cervical cancer. In addition, Cervical adenocarcinoma is characterized by gastric pyloric gland differentiation, and most patients with Cervical adenocarcinoma have a positive expression of Claudin 18.2, so this target is worthwhile for Cervical adenocarcinoma patients. ADCs for other targets are under investigation.

With the continuous progress of biotechnology, the coupling technology of antibody and payload is being updated. At the same time, with the development of radiotherapy, chemotherapy, immunotherapy, and targeted therapy, there are more and more options for developing ADCs combination therapy. Therefore, it is believed that shortly, ADCs are expected to change the traditional treatment of cervical cancer, as well as become the new first- and second-line treatment for patients with recurrent and metastatic cervical cancer.

## Author contributions

CZ: Formal analysis, Conceptualization, Investigation, Methodology, Software, Writing – original draft. YC: Formal analysis, Supervision, Investigation, Methodology, Writing – review & editing. LG: Formal analysis, Investigation, Supervision, Software, Validation, Writing – review & editing. CC: Formal analysis, Conceptualization, Methodology, Writing – review & editing. YS: Conceptualization, Writing – review & editing, Investigation, Software. JZ: Formal analysis, Funding acquisition, Project administration, Resources, Supervision, Writing – review & editing. YW: Formal analysis, Project administration, Resources, Supervision, Writing – review & editing.

## References

[1] Torre LA Bray F Siegel RL Ferlay J Lortet-Tieulent J Jemal A. Global cancer statistics, 2012. CA Cancer J Clin. (2015) 65:87–108. doi: 10.3322/caac.21262 25651787

[2] Endo D Todo Y Okamoto K Minobe S Kato H Nishiyama N. Prognostic factors for patients with cervical cancer treated with concurrent chemoradiotherapy: a retrospective analysis in a Japanese cohort. J Gynecol Oncol. (2015) 26:12–8. doi: 10.3802/jgo.2015.26.1.12 PMC4302279 25310853

[3] Habtemariam LW Zewde ET Simegn GL. Cervix type and cervical cancer classification system using deep learning techniques. Med Devices (Auckl). (2022) 15:163–76. doi: 10.2147/MDER.S366303 PMC9208738 35734419

[4] Bray F Laversanne M Weiderpass E Soerjomataram I. The ever-increasing importance of cancer as a leading cause of premature death worldwide. Cancer. (2021) 127:3029–30. doi: 10.1002/cncr.33587 34086348

[5] Eskander RN Tewari KS. Chemotherapy in the treatment of metastatic, persistent, and recurrent cervical cancer. Curr Opin Obstet Gynecol. (2014) 26:314–21. doi: 10.1097/GCO.0000000000000042 24979076

[6] Tewari KS Sill MW Long HJ Penson RT Huang H Ramondetta LM. Improved survival with bevacizumab in advanced cervical cancer. N Engl J Med. (2014) 370:734–43. doi: 10.1056/NEJMoa1309748 PMC4010094 24552320

[7] Schefter T Winter K Kwon JS Stuhr K Balaraj K Yaremko BP. RTOG 0417: efficacy of bevacizumab in combination with definitive radiation therapy and cisplatin chemotherapy in untreated patients with locally advanced cervical carcinoma. Int J Radiat Oncol Biol Phys. (2014) 88:101–5. doi: 10.1016/j.ijrobp.2013.10.022 24331655

[8] Yin Q Wu L Han L Zheng X Tong R Li L. Immune-related adverse events of immune checkpoint inhibitors: a review. Front Immunol. (2023) 14:1167975. doi: 10.3389/fimmu.2023.1167975 37304306 PMC10247998

[9] Mutlu L Tymon-Rosario J Harold J Menderes G. Targeted treatment options for the management of metastatic/persistent and recurrent cervical cancer. Expert Rev Anticancer Ther. (2022) 22:633–45. doi: 10.1080/14737140.2022.2075348 35533682

[10] Chau CH Steeg PS Figg WD. Antibody-drug conjugates for cancer. Lancet. (2019) 394:793–804. doi: 10.1016/S0140-6736(19)31774-X 31478503

[11] Tarantino P Carmagnani Pestana R Corti C Modi S Bardia A Tolaney SM. Antibody-drug conjugates: Smart chemotherapy delivery across tumor histologies. CA Cancer J Clin. (2022) 72:165–82. doi: 10.3322/caac.21705 34767258

[12] Fu Z Li S Han S Shi C Zhang Y. Antibody drug conjugate: the “biological missile” for targeted cancer therapy. Signal Transduct Target Ther. (2022) 7:93. doi: 10.1038/s41392-022-00947-7 35318309 PMC8941077

[13] Dumontet C Reichert JM Senter PD Lambert JM Beck A. Antibody-drug conjugates come of age in oncology. Nat Rev Drug Discovery. (2023) 22:641–61. doi: 10.1038/s41573-023-00709-2 37308581

[14] Baah S Laws M Rahman KM. Antibody-drug conjugates-A tutorial review. Molecules. (2021) 26:2943. doi: 10.3390/molecules26102943 34063364 PMC8156828

[15] Hoffmann RM Coumbe BGT Josephs DH Mele S Ilieva KM Cheung A. Antibody structure and engineering considerations for the design and function of Antibody Drug Conjugates (ADCs). Oncoimmunology. (2018) 7:e1395127. doi: 10.1080/2162402X.2017.1395127 29375935 PMC5769674

[16] Oh DY Bang YJ. HER2-targeted therapies - a role beyond breast cancer. Nat Rev Clin Oncol. (2020) 17:33–48. doi: 10.1038/s41571-019-0268-3 31548601

[17] Liao S Wang B Zeng R Bao H Chen X Dixit R. Recent advances in trophoblast cell-surface antigen 2 targeted therapy for solid tumors. Drug Dev Res. (2021) 82:1096–110. doi: 10.1002/ddr.21870 34462935

[18] Teicher BA Morris J. Antibody-drug conjugate targets, drugs, and linkers. Curr Cancer Drug Targets. (2022) 22:463–529. doi: 10.2174/1568009622666220224110538 35209819

[19] Jin Y Schladetsch MA Huang X Balunas MJ Wiemer AJ. Stepping forward in antibody-drug conjugate development. Pharmacol Ther. (2022) 229:107917. doi: 10.1016/j.pharmthera.2021.107917 34171334 PMC8702582

[20] Nejadmoghaddam MR Minai-Tehrani A Ghahremanzadeh R Mahmoudi M Dinarvand R Zarnani AH. Antibody-drug conjugates: possibilities and challenges. Avicenna J Med Biotechnol. (2019) 11:3–23. 30800238 PMC6359697

[21] Khongorzul P Ling CJ Khan FU Ihsan AU Zhang J. Antibody-drug conjugates: A comprehensive review. Mol Cancer Res. (2020) 18:3–19. doi: 10.1158/1541-7786.MCR-19-0582 31659006

[22] James BH Papakyriacou P Gardener MJ Gliddon L Weston CJ Lalor PF. The contribution of liver sinusoidal endothelial cells to clearance of therapeutic antibody. Front Physiol. (2021) 12:753833. doi: 10.3389/fphys.2021.753833 35095549 PMC8795706

[23] Song X Li R Wang H Song P Guo W Chen ZS. Tisotumab vedotin for the treatment of cervical carcinoma. Drugs Today (Barc). (2022) 58:213–22. doi: 10.1358/dot.2022.58.5.3400745 35535813

[24] McNamara B Greenman M Pebley N Mutlu L Santin AD. Antibody-drug conjugates (ADC) in HER2/neu-positive gynecologic tumors. Molecules. (2023) 28:7389. doi: 10.3390/molecules28217389 37959808 PMC10650896

[25] Hafeez U Parakh S Gan HK Scott AM. Antibody-drug conjugates for cancer therapy. Molecules. (2020) 25:4764. doi: 10.3390/molecules25204764 33081383 PMC7587605

[26] Birrer MJ Moore KN Betella I Bates RC. Antibody-drug conjugate-based therapeutics: state of the science. J Natl Cancer Inst. (2019) 111:538–49. doi: 10.1093/jnci/djz035 30859213

[27] Lu J Jiang F Lu A Zhang G. Linkers having a crucial role in antibody-drug conjugates. Int J Mol Sci. (2016) 17. doi: 10.3390/ijms17040561 PMC4849017 27089329

[28] Jain N Smith SW Ghone S Tomczuk B. Current ADC linker chemistry. Pharm Res. (2015) 32. doi: 10.1007/s11095-015-1657-7 PMC4596905 25759187

[29] Nolting B. Linker technologies for antibody-drug conjugates. Methods Mol Biol. (2013) 1045:71–100. doi: 10.1007/978-1-62703-541-5_5 23913142

[30] Yoder NC Bai C Tavares D Widdison WC Whiteman KR Wilhelm A. A case study comparing heterogeneous lysine- and site-specific cysteine-conjugated maytansinoid antibody-drug conjugates (ADCs) illustrates the benefits of lysine conjugation. Mol Pharm. (2019) 16:3926–37. doi: 10.1021/acs.molpharmaceut.9b00529 31287952

[31] Joubert N Beck A Dumontet C Denevault-Sabourin C. Antibody-drug conjugates: the last decade. Pharmaceuticals. (2020) 13(9). doi: 10.3390/ph13090245 PMC7558467 32937862

[32] Abdollahpour-Alitappeh M Lotfinia M Gharibi T Mardaneh J Farhadihosseinabadi B Larki P. Antibody-drug conjugates (ADCs) for cancer therapy: Strategies, challenges, and successes. J Cell Physiol. (2019) 234:5628–42. doi: 10.1002/jcp.27419 30478951

[33] Nagayama A Ellisen LW Chabner B Bardia A. Antibody-drug conjugates for the treatment of solid tumors: clinical experience and latest developments. Target Oncol. (2017) 12:719–39. doi: 10.1007/s11523-017-0535-0 29116596

[34] Kalim M Chen J Wang S Lin C Ullah S Liang K. Intracellular trafficking of new anticancer therapeutics: antibody-drug conjugates. Drug Des Devel Ther. (2017) 11:2265–76. doi: 10.2147/DDDT PMC5546728 28814834

[35] Khera E Thurber GM. Pharmacokinetic and immunological considerations for expanding the therapeutic window of next-generation antibody-drug conjugates. BioDrugs. (2018) 32:465–80. doi: 10.1007/s40259-018-0302-5 30132210

[36] Chen YF Xu YY Shao ZM Yu KD. Resistance to antibody-drug conjugates in breast cancer: mechanisms and solutions. Cancer Commun (Lond). (2023) 43:297–337. doi: 10.1002/cac2.12387 36357174 PMC10009672

[37] Loganzo F Tan X Sung M Jin G Myers JS Melamud E. Tumor cells chronically treated with a trastuzumab-maytansinoid antibody-drug conjugate develop varied resistance mechanisms but respond to alternate treatments. Mol Cancer Ther. (2015) 14:952–63. doi: 10.1158/1535-7163.MCT-14-0862 25646013

[38] Drago JZ Modi S Chandarlapaty S. Unlocking the potential of antibody-drug conjugates for cancer therapy. Nat Rev Clin Oncol. (2021) 18:327–44. doi: 10.1038/s41571-021-00470-8 PMC8287784 33558752

[39] Markham A. Tisotumab vedotin: first approval. Drugs. (2021) 81:2141–7. doi: 10.1007/s40265-021-01633-8 34748188

[40] Breij ECW de Goeij BECG Verploegen S Schuurhuis DH Amirkhosravi A Francis J. An antibody-drug conjugate that targets tissue factor exhibits potent therapeutic activity against a broad range of solid tumors. Cancer Res. (2014) 74:1214–26. doi: 10.1158/0008-5472.CAN-13-2440 24371232

[41] Martín-Sabroso C Lozza I Torres-Suárez AI Fraguas-Sánchez AI. Antibody-antineoplastic conjugates in gynecological Malignancies: current status and future perspectives. Pharmaceutics. (2021) 13:1705. doi: 10.3390/pharmaceutics13101705 34683998 PMC8541375

[42] Hisada Y Mackman N. Tissue factor and cancer: regulation, tumor growth, and metastasis. Semin Thromb Hemost. (2019) 45:385–95. doi: 10.1055/s-0039-1687894 PMC6546519 31096306

[43] de Goeij BECG Satijn D Freitag CM Wubbolts R Bleeker WK Khasanov A. High turnover of tissue factor enables efficient intracellular delivery of antibody-drug conjugates. Mol Cancer Ther. (2015) 14:1130–40. doi: 10.1158/1535-7163.MCT-14-0798 25724665

[44] Seidel UJE Schlegel P Lang P. Natural killer cell mediated antibody-dependent cellular cytotoxicity in tumor immunotherapy with therapeutic antibodies. Front Immunol. (2013) 4:76. doi: 10.3389/fimmu.2013.00076 23543707 PMC3608903

[45] de Bono JS Concin N Hong DS Thistlethwaite FC Machiels JP Arkenau HT. Tisotumab vedotin in patients with advanced or metastatic solid tumours (InnovaTV 201): a first-in-human, multicentre, phase 1–2 trial. Lancet Oncol. (2019) 20:383–93. doi: 10.1016/S1470-2045(18)30859-3 30745090

[46] Coleman RL Lorusso D Gennigens C González-Martín A Randall L Cibula D. Efficacy and safety of tisotumab vedotin in previously treated recurrent or metastatic cervical cancer (innovaTV 204/GOG-3023/ENGOT-cx6): a multicentre, open-label, single-arm, phase 2 study. Lancet Oncol. (2021) 22:609–19. doi: 10.1016/S1470-2045(21)00056-5 33845034

[47] Vergote I Van Nieuwenhuysen E O’Cearbhaill RE Westermann A Lorusso D Ghamande S. Tisotumab vedotin in combination with carboplatin, pembrolizumab, or bevacizumab in recurrent or metastatic cervical cancer: results from the innovaTV 205/GOG-3024/ENGOT-cx8 study. J Clin Oncol. (2023) 41:5536–49. doi: 10.1200/JCO.23.00720 PMC10730069 37651655

[48] Bogani G Coleman RL Vergote I Raspagliesi F Lorusso D Monk BJ. Tisotumab vedotin in recurrent or metastatic cervical cancer. Curr Problems Cancer. (2023) 47:100952. doi: 10.1016/j.currproblcancer.2023.100952 36842202

[49] Nakada T Sugihara K Jikoh T Abe Y Agatsuma T. The latest research and development into the antibody-drug conjugate, [fam-] trastuzumab deruxtecan (DS-8201a), for HER2 cancer therapy. Chem Pharm Bull (Tokyo). (2019) 67:173–85. doi: 10.1248/cpb.c18-00744 30827997

[50] Ogitani Y Aida T Hagihara K Yamaguchi J Ishii C Harada N. DS-8201a, A novel HER2-targeting ADC with a novel DNA topoisomerase I inhibitor, demonstrates a promising antitumor efficacy with differentiation from T-DM1. Clin Cancer Res. (2016) 22:5097–108. doi: 10.1158/1078-0432.CCR-15-2822 27026201

[51] Doi T Shitara K Naito Y Shimomura A Fujiwara Y Yonemori K. Safety, pharmacokinetics, and antitumour activity of trastuzumab deruxtecan (DS-8201), a HER2-targeting antibody-drug conjugate, in patients with advanced breast and gastric or gastro-oesophageal tumours: a phase 1 dose-escalation study. Lancet Oncol. (2017) 18:1512–22. doi: 10.1016/S1470-2045(17)30604-6 29037983

[52] Shitara K Iwata H Takahashi S Tamura K Park H Modi S. Trastuzumab deruxtecan (DS-8201a) in patients with advanced HER2-positive gastric cancer: a dose-expansion, phase 1 study. Lancet Oncol. (2019) 20:827–36. doi: 10.1016/S1470-2045(19)30088-9 31047804

[53] Nishikawa T Hasegawa K Matsumoto K Mori M Hirashima Y Takehara K. Trastuzumab deruxtecan for human epidermal growth factor receptor 2-expressing advanced or recurrent uterine carcinosarcoma (NCCH1615): the STATICE trial. J Clin Oncol. (2023) 41:2789–99. doi: 10.1200/JCO.22.02558 PMC10414746 36977309

[54] Meric-Bernstam F Makker V Oaknin A Oh DY Banerjee S González-Martín A. Efficacy and safety of trastuzumab deruxtecan in patients with HER2-expressing solid tumors: primary results from the DESTINY-panTumor02 phase II trial. J Clin Oncol. (2024) 42:47–58. doi: 10.1200/JCO.23.02005 37870536 PMC10730032

[55] Xu Y Wang Y Gong J Zhang X Peng Z Sheng X. Phase I study of the recombinant humanized anti-HER2 monoclonal antibody-MMAE conjugate RC48-ADC in patients with HER2-positive advanced solid tumors. Gastric Cancer. (2021) 24:913–25. doi: 10.1007/s10120-021-01168-7 PMC8205919 33945049

[56] Zaman S Jadid H Denson AC Gray JE. Targeting Trop-2 in solid tumors: future prospects. Onco Targets Ther. (2019) 12:1781–90. doi: 10.2147/OTT PMC6402435 30881031

[57] Starodub AN Ocean AJ Shah MA Guarino MJ Picozzi VJ Vahdat LT. First-in-human trial of a novel anti-trop-2 antibody-SN-38 conjugate, sacituzumab govitecan, for the treatment of diverse metastatic solid tumors. Clin Cancer Res. (2015) 21:3870–8. doi: 10.1158/1078-0432.CCR-14-3321 PMC4558321 25944802

[58] Zeybek B Manzano A Bianchi A Bonazzoli E Bellone S Buza N. Cervical carcinomas that overexpress human trophoblast cell-surface marker (Trop-2) are highly sensitive to the antibody-drug conjugate sacituzumab govitecan. Sci Rep. (2020) 10:973. doi: 10.1038/s41598-020-58009-3 31969666 PMC6976591

[59] Bouleftour W Guillot A Magne N. The anti-nectin 4: A promising tumor cells target. A Systematic Review. Mol Cancer Ther. (2022) 21:493–501. doi: 10.1158/1535-7163.MCT-21-0846 35131876

[60] Wong JL Rosenberg JE. Targeting nectin-4 by antibody-drug conjugates for the treatment of urothelial carcinoma. Expert Opin Biol Ther. (2021) 21:863–73. doi: 10.1080/14712598.2021.1929168 PMC8224177 34030536

[61] Zhou W Fang P Yu D Ren H You M Yin L. Preclinical evaluation of 9MW2821, a site-specific monomethyl auristatin E-based antibody-drug conjugate for treatment of nectin-4-expressing cancers. Mol Cancer Ther. (2023) 22:913–25. doi: 10.1158/1535-7163.MCT-22-0743 PMC10390865 37196158

[62] Cao W Xing H Li Y Tian W Song Y Jiang Z. Claudin18.2 is a novel molecular biomarker for tumor-targeted immunotherapy. biomark Res. (2022) 10:38. doi: 10.1186/s40364-022-00385-1 35642043 PMC9153115

[63] Yan P Dong Y Zhang F Zhen T Liang J Shi H. Claudin18.2 expression and its clinicopathological feature in adenocarcinoma from various parts. J Clin Pathol. (2024), 2023-209268. doi: 10.1136/jcp-2023-209268 PMC12703292 38548320

